# Fluorescence quenching-based immunological probe for ticagrelor monitoring

**DOI:** 10.3389/fbioe.2023.1295406

**Published:** 2023-11-28

**Authors:** Shengshuo Zhang, Yueqing Cheng, Yujie Gao, Yujie Zou, Weiling Xiao, Tianyi Li, Mei Li, Bowen Yu, Jinhua Dong

**Affiliations:** ^1^ School of Life Science and Technology, Weifang Medical University, Weifang, China; ^2^ School of Rehabilitation Sciences and Engineering, University of Health and Rehabilitation Sciences, Qingdao, China; ^3^ School of Basic Medical Sciences, Weifang Medical University, Weifang, China; ^4^ School of Stomatology, Weifang Medical University, Weifang, China; ^5^ International Research Frontiers Initiative, Tokyo Institute of Technology, Yokohama, Japan

**Keywords:** ticagrelor, acute coronary syndrome, Q-body, immunofluorescence sensor, rapid detection, full-length IgG

## Abstract

**Introduction:** Ticagrelor is extensively utilized for the treatment of acute coronary syndromes (ACS), but its platelet aggregation inhibitory effects can potentially result in tissue bleeding, posing a serious risk to patients’ lives.

**Methods:** In this study, we developed highly sensitive full length anti-ticagrelor Quenchbodies (Q-bodies) for fast monitoring of ticagrelor both in solution and serum for the first time. Ticagrelor coupled with N- hydroxysuccinimide (Ticagrelor-NHS) ester was also designed and synthesized for interaction and biological activity detection.

**Results:** Both ATTO-labeled MEDI2452 (2452A) Q-body and TAMRA-labeled IgG 152 (152T) Q-body demonstrated efficient detection of ticagrelor and its active metabolite (TAM). The 2452A Q-body exhibited a broader detection range, while the 152T Q-body displayed a lower limit of detection (LOD). Under physiological conditions (Ticagrelor:TAM, 3:1), the concentration of ticagrelor was further measured, yielding LOD values of 4.65 pg/mL and 2.75 pg/mL for the two Q-bodies, with half-maximal effect concentrations of 8.15 ng/mL and 3.0 ng/mL, respectively.

**Discussion:** Compared with traditional liquid chromatography-mass spectrometry (LC-MS) methods, anti-ticagrelor Q-bodies have higher sensitivity and detection speed. It enabled the completion of analysis within 3 min, facilitating rapid preoperative detection of blood drug concentration in ACS to determine the feasibility of surgery and mitigate the risk of intraoperative and postoperative hemorrhage. The swift detection of ticagrelor holds promise for enhancing individualized drug administration, preventing adverse reactions, and providing preoperative guidance.

## 1 Introduction

Acute coronary syndrome (ACS) is a common disease prevalent worldwide, and although its lethality has declined in the last 30 years, it remains the leading cause of human death with high morbidity and mortality (Chan et al., 2016; Bergmark et al., 2022). The main treatment for ACS is antiplatelet coagulation (Shifrin and Widmar, 2016; Tsoumani et al., 2016; Szarpak et al., 2022). Ticagrelor, currently the most commonly used antiplatelet coagulation inhibitor, is widely employed in ACS treatment (Kabil et al., 2022; Tao et al., 2022). It acts as a selective and reversible adenosine diphosphate (ADP) receptor antagonist on the platelet ADP receptor P2Y_12_, inhibiting ADP-mediated platelet activation and aggregation (Guerbaai et al., 2019; Akkaif et al., 2021; Sanderson et al., 2021). Ticagrelor metabolism produces various metabolites, but only AR-C124910XX, known as Ticagrelor Active Metabolite (TAM), exhibits similar potency to ticagrelor and is present in the patient’s circulation at approximately one-third of the parent drug’s concentration (Akkaif et al., 2020). The other metabolites, referred to as Ticagrelor Inactive Metabolite (TIM), do not inhibit the P2Y_12_ receptor (Buchanan et al., 2015). Following oral administration of 200 mg of ticagrelor, the maximum blood concentrations of ticagrelor and TAM are reached at 860 ng/mL and 240 ng/mL, respectively, after 6 h. Both ticagrelor and TAM are gradually metabolized and completely excreted within the subsequent 42 h (Teng et al., 2010). Being antiplatelet agents, ticagrelor medications carry an increased risk of spontaneous or postoperative bleeding, necessitating close clinical monitoring of blood levels (Angheloiu et al., 2017; Guerbaai et al., 2019; Ennezat et al., 2022; Yang et al., 2022). Currently, liquid chromatography-mass spectrometry (LC-MS) has become the most common method for the detection of ticagrelor and TAM in organisms in preclinical and clinical studies (Chae et al., 2019; Kelemen et al., 2019). While LC-MS analysis provides accurate results, it is complex, time-consuming, and expensive to operate. In contrast, immunological detection offers advantages such as not requiring extensive equipment, simplicity, ease of implementation, and good sample selectivity. Representative techniques include immunofluorescence detection and enzyme-linked immunosorbent assay (ELISA). However, some of these techniques are complicated to operate and time-consuming. Additionally, the sensitivity and specificity of these methods may vary depending on the specific antigen and antibody employed. Sandwich ELISA, a highly sensitive method, is unable to effectively detect monovalent antigens with a molecular weight less than 1000, primarily due to their small size. In such cases, competitive ELISA is usually employed. However, it is generally less sensitive than sandwich ELISA due to the ratiometric principle that requires the use of only one antibody (Ueda, 2007; Aydin, 2015). Q-body, a fluorescence-quenching-based immunosensor, is a homogeneous immunofluorescence detection technique known for its high sensitivity, accuracy, and efficiency (Abe et al., 2011; Dong and Ueda, 2021). By labeling the variable region of the antibody with a fluorescent dye, the dye enters the antigen-binding region, and the internal tryptophan of the antibody provides electrons through an indole group, mediating photo-induced electron transfer (PET), resulting in quenching of the fluorescent dye (Ohashi et al., 2016). When the Q-body binds to the antigen, the fluorescent dye is released, leading to an increase in fluorescence intensity. This change in fluorescence intensity can be used to indicate the antigen’s concentration (Abe et al., 2014; Jeong et al., 2018). Q-body can be used for the detection of not only macromolecules such as proteins but also small molecules such as hormones, hazardous chemicals, and peptides (Wood et al., 2007; Dong et al., 2018; Zhao et al., 2018). While antigen-binding fragments (Fab) have been widely used to prepare various Q-bodies, a successful preparation of full-length IgG-based Q-bodies has also been reported for detecting the FLAG tag (Gao et al., 2023).

A ticagrelor-affinity Fab 72 antibody fragment and its mutants, Fab 152, Fab 162, and Fab MEDI2452, with high affinity for ticagrelor and TAM, have been reported previously (Buchanan et al., 2015). In this study, we prepared and thoroughly investigated eight full-length anti-ticagrelor Q-bodies for highly sensitive detection of ticagrelor and its metabolite. Additionally, we designed and synthesized a ticagrelor-NHS ester capable of covalently labeling proteins to detect antibody interactions and tested the potential drug formation of albumin-coupled ticagrelor.

## 2 Materials and methods

### 2.1 Materials

*E. coli* DH5α for gene cloning was purchased from Agilent (La Jolla, CA, USA). Restriction endonucleases were purchased from New England Biolabs (Beverly, MA, USA). HiFi high-fidelity PCR mix was purchased from Yongke Biotech Co., Ltd (Shanghai, China). Homologous cloning enzyme was purchased from Vazyme Biotech Co., Ltd (Nanjing, China). Agarose was purchased from Biofroxx (Einhausen, Germany). FITC-conjugated PAC-1 (340507) was purchased from BD Biosciences (San Jose, CA, USA). Tris (2-carboxyethyl) phosphine (TCEP) was purchased from Thermo Pierce (Rockford, IL, USA). The fluorescent dye 5(6)- carboxytetramethylrhodamine-C6-maleimide (TAMRA) was purchased from Lumiprobe Corporation (Hunt Valley, MD, USA). ATTO520-C2-maleimide (ATTO) was purchased from ATTO-TEC GmbH (Siegen, GA, USA). rProteinA Beads were purchased from Changzhou Tiandi Renhe Biotechnology Co., Ltd (Changzhou, China). Isopropyl β-D-thiogalactopyranoside (IPTG), bovine serum albumin (BSA), and Ni-NTA Sefinose™ Resin were obtained from Shanghai Sangon Biotechnology Co., Ltd (Shanghai, China). Coomassie brilliant blue R-250 was purchased from Beijing Solabio Technology Co., Ltd (Beijing, China); linearized polyethyleneimine transfection reagent (PEI) was purchased from Shanghai Maokang Biotechnology Co., Ltd (Shanghai, China) and ticagrelor was purchased from Shanghai Aladdin Biochemical Science & Technology Co., Ltd (Shanghai, China). Digoxin was purchased from Chengdu Alfa Biotechnology Co., Ltd (Chengdu, China). TAM and TIM were purchased from Shanghai Biochempartner Co., Ltd (Shanghai, China).

### 2.2 Construction of full-length antibody IgG eukaryotic expression vector

The gene coding heavy chain variable and constant region 1 (V_H_-C_H1_) and the light chain variable and constant region (V_L_-C_L_) of IgG MEDI2452 were amplified by PCR using primers 5ALBHfor/5ALBHback and 5ALBLfor/5ALBLback, respectively. The gene for heavy chain constant regions 2 and 3 (C_H2_-C_H3_; Fc) was amplified by using primers 5ALBFcfor and 5ALBFcback. The amplified DNA products V_H_-C_H1_ and Fc were then linked by an overlap PCR and mixed with the *Bam*HI/*Xho*I double-digested vector pMlink and linked by using homologous recombinase. Plasmid with the right sequences was designated as pMlink-5ALB-H. The light chain was ligated to the double-digested vector pMlink by homologous recombination with the above method and named pMlink-5ALB-L (Alexandrov et al., 2004). The primers 5ALBMutantLfor/5ALBMutantLback and 5ALBMutantHfor/5ALBMutantHback were used to replace several bases in the heavy and light chains. The vectors were named pMlink-5ALB-HM and pMlink-5ALB-LM, respectively. The primers used are summarized in Supplementary Table S1.

### 2.3 Expression and purification of full-length antibodies

HEK293F cells were utilized to express full-length antibodies. MEDI2452 antibody was expressed by mixing pMlink-5ALB-H and pMlink-5ALB-L with PEI and allowed to stand for 15 min. The mixture was added to cell suspension with a density of 1.5 × 10^6^ cells/mL and incubated at 37°C, 8% CO_2_, 150 rpm on a shaker for 96 h. Antibodies IgG 72, IgG 152, and IgG 162 were expressed similarly. After expression, the cell suspension was centrifuged at 13,000×g for 20 min, and the supernatant was extracted and combined with 500 μL of ProteinA beads for 2 h. The beads were washed with 10 mL of PBS, and then eluted with 7 mL of 0.1 M Glycine at pH 2.8 and neutralized with 700 μL of 1.5 M Tris-HCl at pH 8.8; the eluted protein solution was buffer changed into PBS through ultrafiltration. The four antibodies obtained from purification were analyzed by SDS-PAGE to determine their molecular size and purity. After separation, SDS-PAGE was stained with Coomassie Brilliant Blue staining (CBB) solution and observed under a Tanon-5200Multi gel image analyzer.

### 2.4 Design and synthesis of ticagrelor-NHS ester and coupling to BSA

For the design of the ticagrelor-NHS ester, a 6C link was attached via an ester group to the hydroxyl group of ticagrelor away from the C ring and a succinimidyl lactone via an ester group on the other side of the link. Ticagrelor-NHS ester was synthesized by Wuxi Apptec (Tianjin) Co., Ltd. Ticagrelor-NHS ester was coupled with BSA according to the following steps. Briefly, 50 μL of 2.25 mg/mL ticagrelor-NHS ester in DMSO solution was mixed with 450 μL 1.11 mg/mL BSA in PBS (pH 8.0) and allowed to stand on ice for 2 h. At the end of the coupling, 4 mL of TBS buffer was added to terminate the reaction, and the unlabeled ticagrelor-NHS ester was removed by ultrafiltration. The prepared BSA-Ticagrelor (BSA-Tic) was analyzed by SDS-PAGE to determine its molecular size and purity.

### 2.5 Platelet agglutination inhibition test

The platelet agglutination inhibition test was performed following previously described methods (Zhao et al., 2017; Zhang et al., 2020; Polasky et al., 2021). Platelet-rich plasma (PRP) was prepared from citrated whole blood (1:7 v/v) by centrifugation at 300×g for 11 min. PRP (100 μL, 3×10^8^/mL) was incubated with DMSO, ticagrelor, or BSA-Tic at a final concentration of 1.25 μg/mL for 20 min. Subsequently, platelet activation was induced by 20 mM ADP. Platelet activation was detected by measuring integrin αIIbβ3 activation. Activated GPIIb/IIIa was detected by FITC-labeled mouse anti-human PAC-1 antibody (BD Biosciences) that specifically recognizes αIIbβ3 of activated platelets. Platelets were analyzed by flow cytometry (BD FACS AriaIII).

### 2.6 Q-body preparation

The four purified antibodies, namely, MDE2452, IgG 72, IgG 152, and IgG 162, were labeled with ATTO520-C2-maleimide and 5(6)-TAMRA-C6-maleimide fluorescent dyes under light protection. To reduce the Cys residue on the N-terminal tag of the antibody heavy and light chain, 100 μL of each antibody, diluted to 1 mg/mL, was mixed with 2 μL of 0.1 M TCEP, respectively. After incubation on a vertical shaker at 4°C for 20 min, the reaction was terminated by adding 4-azidobenzoic acid solution at a final concentration of 2 mM and placing it on ice for 10 min (Henkel et al., 2016). Subsequently, ATTO520-C2-maleimide or 5(6)-TAMRA-C6-maleimide was added to the respective reactions. Labeled antibodies were detected by SDS-PAGE, and the fluorescent images were obtained and finally stained with CBB.

### 2.7 ELISA

The enzyme plate was coated with 100 μL of BSA-Tic at a concentration of 5 μg/mL and incubated at 4°C overnight. Each well was then blocked with 200 μL of a 2% skim milk powder solution in PBS for 2 h, followed by three washes with PBST (PBS containing 0.1% Tween-20). Next, 100 μL of antibody or Q-body at a concentration of 10 μg/mL was added to each well and incubated at room temperature for 1 h. After three washes with PBST, 100 μL of 1:3000 dilution of horseradish peroxidase-labeled rabbit anti-human antibody was added and incubated for 1 h at 25°C, followed by six washes with PBST. The reaction was visualized by adding 100 μL of 3,3′,5,5′-tetramethylbenzidine solution to each well, and the reaction was terminated by adding 50 μL of 10% H_2_SO_4_. The absorbance at 450 nm, with a reference wavelength of 630 nm was measured using an iMark™ microplate reader.

### 2.8 Fluorescence measurements

Denaturants were used to determine the initial quenching degree of the Q-body. Equal amounts of Q-body were added to 1 mL of denaturant (7M GdnHCl, 100 mM DTT in PBST), and the fluorescence intensity was scanned on a Hitachi F-4600 fluorescence spectrophotometer. The scanning range was 535–700 nm for ATTO and 565–700 nm for TAMRA, with excitation wavelengths of 520 or 546 nm. For antigen detection, a final concentration of 50 ng/mL of ticagrelor was added to PBS or spiked serum (PBS containing 10% serum) along with the anti-ticagrelor Q-body. The fluorescence intensity was scanned each minute. For the detection of the gradient-diluted ticagrelor, 1 μL of gradient-diluted ticagrelor (0.5 ng/mL–5 mg/mL) was added to PBS or spiked serum. After incubation at room temperature for 3 min, the fluorescence intensity was measured. This procedure was repeated three times. TAM, TIM, digoxin, and ticagrelor:TAM (3:1) were detected similarly compared to ticagrelor.

### 2.9 Data treatment and statistical analysis

All the experiments were performed at least three times. Statistical analyses were performed using Prism 8.0 (GraphPad Software, San Diego, CA, USA). Dose-response curves were fitted to a four-parameter logistic equation y = d+ (a-d)/(1+(x/c)^b^) in order to determine the half-maximal effect concentration (EC50) and the lowest limit of detection (LOD). The LOD value represented the estimated antigenic concentration, which was calculated as the mean blank fluorescence intensity plus three times the standard deviation (*n* = 3). A *p*-value <0.05 was considered statistically significant and denoted by an asterisk (*); similarly, *p* < 0.01 was denoted as a double asterisk (**), and *p* < 0.001 was denoted as a triple asterisk (***). The multiplicative fluorescence increases of 0.3 ng/mL, 3 ng/mL, and 30 ng/mL were brought into a four-parameter logarithmic equation to calculate the recovery rate of spiked ticagrelor:TAM (3:1) in human serum.

## 3 Results

### 3.1 Design and construction of full-length IgG expression vectors

**FIGURE 1:**
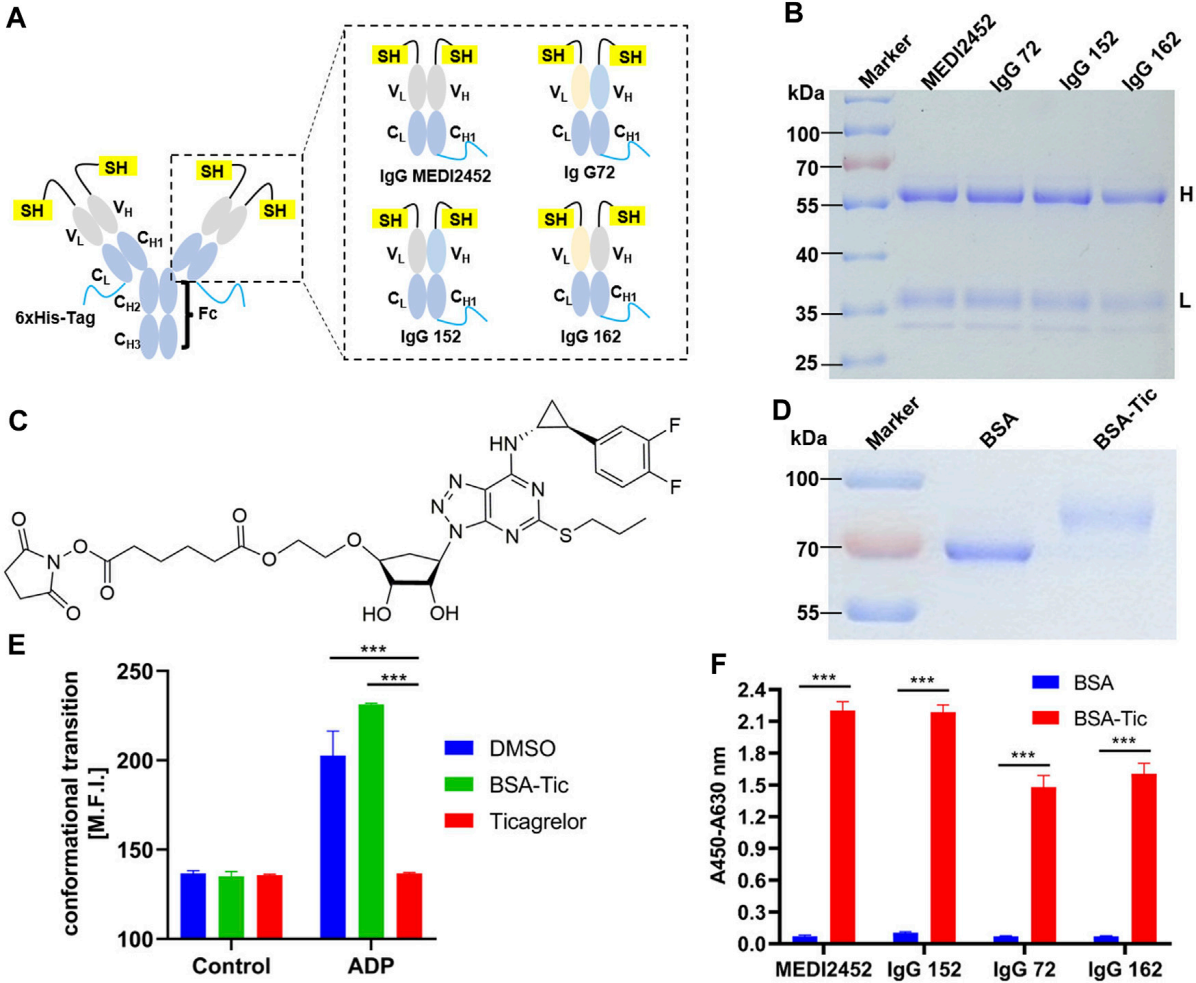
Antibody expression and activity assay. **(A)** Schematic structure of MEDI2452, IgG 72, IgG 152, and IgG 162; **(B)** SDS-PAGE coomassie brilliant blue after antibody expression purification; **(C)** Structure of ticagrelor-NHS ester; **(D)** SDS-PAGE coomassie brilliant blue of ticagrelor-NHS ester labeled with BSA after purification; **(E)** Mean fluorescence intensity of FACS of PAC1 to detect the inhibitory effect of BSA-Tic on platelets; **(F)** ELISA to detect the binding activity of antibody to BSA-Tic. Adjusted *p*-values in Panels **(E,F)** were calculated by one-way ANOVA with Tukey’s multiple comparisons test. H: antibody heavy chain fragment; L: antibody light chain fragment; Error bars, ±1 S.D (*n* = 3); ***: *p* < 0.001.

Four Fab fragments-binding ticagrelor, namely, Fab MEDI2452, Fab 72, Fab 152, and Fab 162 which share identical light or heavy chains have been previously reported (Buchanan et al., 2015) (Figure 1A). In comparison to Fab, the full-length IgG Q-body was more stable and easier to purify (Gao et al., 2023). Therefore, we constructed eukaryotic expression vectors to express Fab MEDI2452, Fab 72, Fab 152, and Fab 162 in the form of IgG in HEK293F cells. We added a 6×His-Tag at the C-terminal end of the light chain to confirm the existence of the light chain and purification after labeling. Additionally, a Cys-Tag peptide (MAQIEVNCSNET) was added at the N-terminal end of the heavy chain and another with the sequence of MSKQCSNET was added to the light chains for fluorescent dye modification (Figure 1A).

### 3.2 Preparation of MEDI2452, IgG 72, IgG 152, and IgG 162 antibodies

The purified MEDI2452, IgG 72, IgG 152, or IgG 162 antibodies were analyzed using SDS-PAGE. After CBB staining, two bands representing the heavy and light chains were observed at approximately 60 kDa and 37 kDa, respectively, for each of the four antibodies. The size of the four antibodies was consistent (Figure 1B). Since only a few amino acids were replaced in the heavy and light chains of the four antibodies, their size remained largely unchanged.

### 3.3 Ticagrelor-NHS ester coupled BSA and platelet agglutination test

Since the small molecule drug ticagrelor was not available for immobilization onto ELISA plate, we designed Ticagrelor-NHS ester (Figure 1C). SDS-PAGE result showed that BSA and the coupling product BSA-Tic were approximately 66 kDa and 75 kDa, respectively (Figure 1D). The larger molecular size of BSA-Tic confirmed that Ticagrelor-NHS ester had been labeled onto BSA. We further test whether BSA-Tic has a similar inhibitory effect compared to ticagrelor on platelet aggregation which makes BSA-Tic a potential protein-conjugated drug. Therefore, platelet agglutination inhibition assays were performed, and platelet activation was detected by integrin αIIbβ3 activation. Activated GPIIb/IIIa was detected by FITC-labeled PAC-1 binding platelets (Manohar et al., 2021) (Figure 1E). The mean fluorescence intensity of the platelets incubated with DMSO, ticagrelor, and BSA-Tic in the control group was 136.7, 135.7, and 135, respectively. The DMSO group increased from 136.7 to 202.7, indicating that platelets were activated. The ticagrelor group was essentially unchanged, indicating that ticagrelor inhibited platelet activation. The BSA-Tic group increased from 135 to 231.3, indicating that BSA-Tic did not inhibit platelet activation (Figure 1E). The undetected activation activity of BSA-Tic may be due to the spatial hindrance caused by structural changes after ticagrelor was bound to BSA and prevented it from binding properly to the ADP-binding site of P2Y_12_. These results suggest that BSA-Tic may not be a potential protein-coupled small-molecule drug. However, it can still serve as a suitable tool for detecting interactions between antibodies and small molecules.

### 3.4 Preparation of Q-body

MEDI2452, IgG 72, IgG 52, and IgG 162 were verified for their binding activity to BSA-Tic by ELISA. The purified antibodies were able to bind BSA-Tic but not BSA, demonstrating their specific binding activity for ticagrelor. In addition, MEDI2452 and IgG 152 exhibited better binding ability to BSA-Tic compared to IgG 72 and IgG 162 (Figure 1F), which is consistent with previously reported results (Buchanan et al., 2015).

**FIGURE 2:**
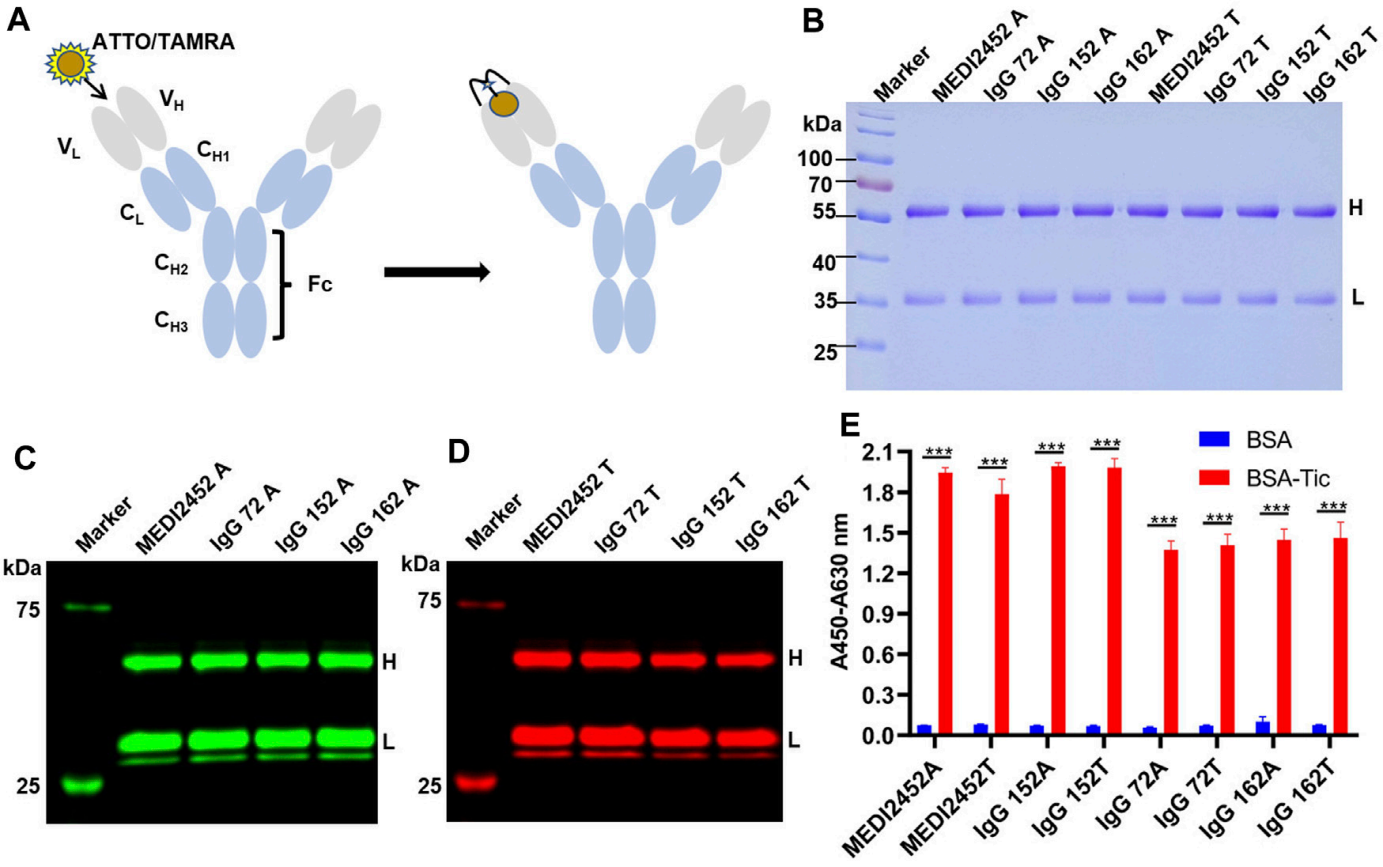
Preparation of Anti-ticagrelor Q-body and its activity detection. **(A)** Schematic diagram of ATTO/TAMRA-labeled antibody; **(B)** SDS-PAGE coomassie brilliant blue of fluorescently labeled anti-ticagrelor Q-body; **(C)** Fluorescence imaging of ATTO-labeled antibody; **(D)** Fluorescence imaging of TAMRA-labeled antibody; **(E)** ELISA detection of BSA-Tic binding activity of fluorescently labeled Q-body. Adjusted *p*-values in Panel **(E)** were calculated by one-way ANOVA with Tukey’s multiple comparisons test. H: Antibody heavy chain fragment; L: Antibody light chain fragment; Error bars, ±1 S.D (*n* = 3); ***: *p* < 0.001.

The maleimide moiety of the fluorescent dye reacts with the N-terminal Cys-Tag group of the antibody, resulting in the labeling of the dye on Cys (Lane et al., 2018). In this study, the fluorescent dyes ATTO and TAMRA were used to label MEDI2452, IgG 72, IgG 152, and IgG 162, respectively (Figure 2A). To remove the free fluorescent dye, the labeled antibody (anti-ticagrelor Q-body) was purified with Ni-NTA resin. The Ni-NTA beads specifically bind to the 6× His-tag, which is located at the C-terminus of the light chain, ensuring the presence of the light chain. The purified Q-body was analyzed by SDS-PAGE, and the CBB staining images also showed their high purity (Figure 2B). The fluorescence imaging showed that both heavy and light chains were successfully labeled with ATTO (Figure 2C) or TAMRA (Figure 2D). The antigen-binding activity of Q-bodies was tested by ELISA, and the results showed that they had antigen-binding activity similar to that of unlabeled antibody, indicating that fluorescent labeling does not affect the activity of antibody (Figure 2E).

### 3.5 Denaturation assay

**FIGURE 3:**
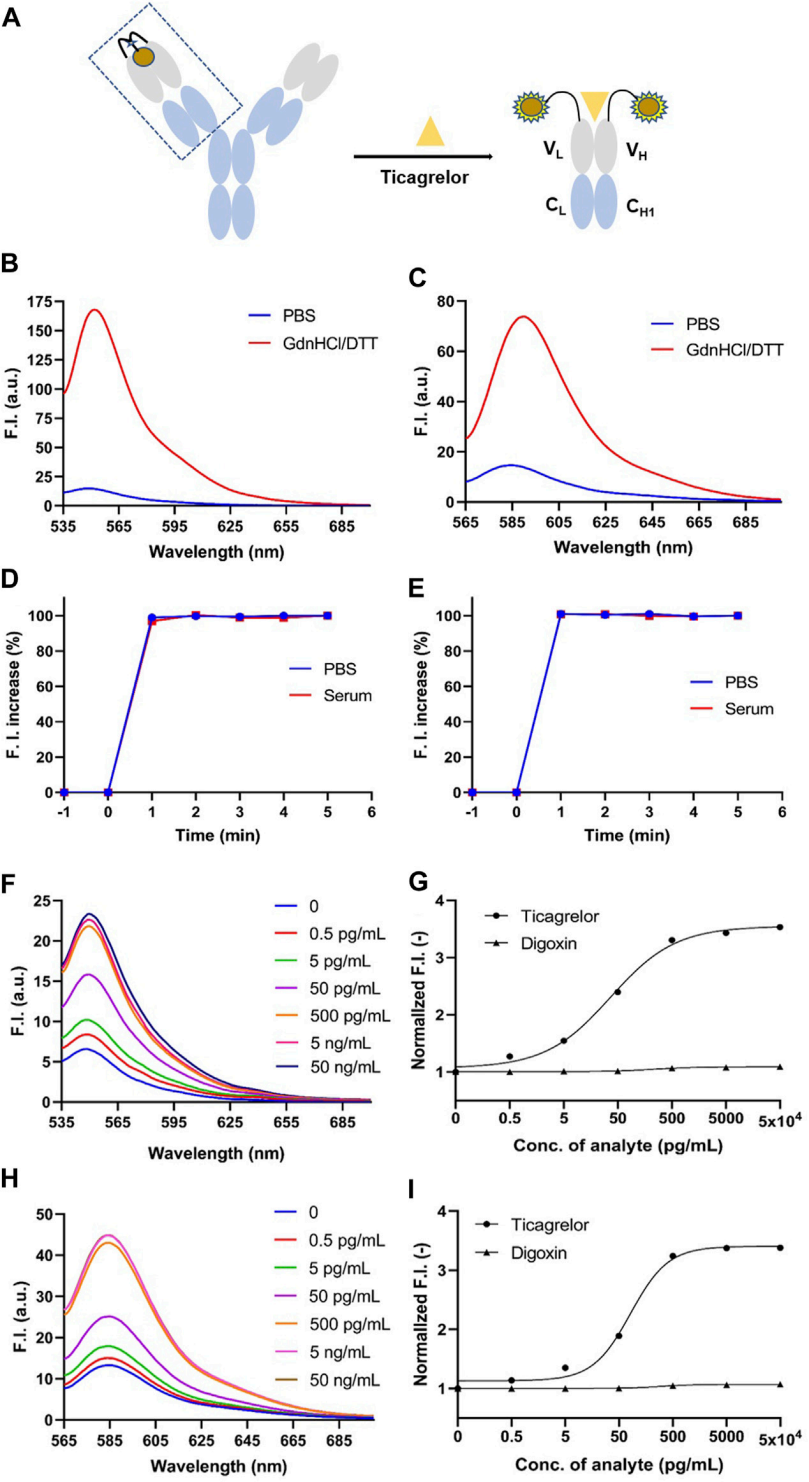
GdnHCl/DTT denaturation assay of anti-ticagrelor Q-body and detection of ticagrelor and digoxin in PBS. **(A)** Schematic diagram of Q-body detection of ticagrelor; **(B)** 2452A Q-body denaturation assay spectrum in GdnHCl/DTT denaturant; **(C)** 152T Q-body denaturation assay spectrum in GdnHCl/DTT denaturant. **(D)** Time-stability curves of 2452A Q-body for the detection of ticagrelor in PBS and serum; **(E)** Time-stability curves of 152T Q-body for the detection of ticagrelor in PBS and serum; **(F)** Spectra of 2452A Q-body after addition of gradient-diluted ticagrelor with PBS as background; **(G)** Stoichiometric-response curves for detection of ticagrelor and digoxin in PBS by 2452A Q-body; **(H)** Spectra of 152T Q-body after addition of gradient-diluted ticagrelor with PBS as background; **(I)** Stoichiometric-response curves for detection of ticagrelor and Digoxin in PBS by 152T Q-body. F.I: fluorescence intensity; GdnHCl/DTT: 7 M guanidine hydrochloride, 100 mM dithiothreitol; Error bars, ±1 S.D (*n* = 3).

In the absence of antigen, the fluorescent dye interacts with the tryptophan on the V_H_ and V_L_ of the Q-body via maleimide, leading to quenching. However, when the Q-body bonded to the antigen, the fluorescent dye moved from the antibody and recovered its fluorescence (Figure 3A). By using GdnHCl/DTT as a denaturant, the original conformation of the antibody’s variable region is disrupted, causing the tryptophan residues to be unable to provide electrons. This release of fluorescence restores the fluorescence intensity of the fluorescent dye (Dong and Ueda, 2021). Equal amounts of Q-body were added to equal amounts of PBS and GdnHCl/DTT, respectively, and the degree of initial quenching of Q-body was detected. The spectrum for ATTO-labeled 2452A Q-body and TAMRA-labeled 152T Q-body showed that the fluorescent intensity increased 11.32-fold and 5.06-fold, respectively (Figures 3B, C) which were highest among all labeled antibodies; in addition, all Q-bodies in the GdnHCl/DTT were all higher in fluorescence intensity than in PBS (Supplementary Table S2). The higher fluorescence increased Q-bodies labeled by MEDI2452 and IgG125, possibly due to the presence of more aromatic amino acids Tyrosine in both heavy and light chains (Abe et al., 2011; Dong and Ueda, 2021) (Supplementary Tables S2, S3). These results show that the Q-body may be able to detect the concentration of ticagrelor by detecting the change in fluorescence intensity upon antigen-antibody binding. Among them, 2452A and 152T were further chosen for sensitivity detection of ticagrelor.

We added 50 ng/mL ticagrelor to PBS or spiked serum with anti-ticagrelor Q-body and detected the change in fluorescence intensity over time. Both 2452A and 152T Q-body reached a steady state at 1 min in PBS or spiked serum (Figures 3D, E). To ensure the accuracy of the experimental data, we performed the fluorescence assay after adding the antigen in the subsequent assay and incubating it for 3 min at room temperature.

### 3.6 Detection of ticagrelor and TAM in PBS

The detection of ticagrelor in aqueous solutions can be applied to determine the final product concentration in pharmaceutical processes and identify drug concentrations in ticagrelor-related scientific research. Firstly, gradient dilutions of ticagrelor or TAM were added to PBS containing Q-body. The antigen concentration started as low as 0.5 pg/mL and increased in a 10-fold gradient. The fluorescence intensity no longer increased when ticagrelor was added to 50 ng/mL, which may be due to the limited solubility of ticagrelor in PBS. The fluorescence intensities of the 2452A and 152T Q-body approached their highest when the ticagrelor reached 5 ng/mL (Figures 3F, H). The excitation wavelength for 2452A Q-body was 520 nm, and the maximum fluorescence peak of the emission wavelength appeared at 549 nm, corresponding to the ATTO fluorescent dye (Figure 3F). The excitation wavelength for the TAMRA-labeled 152T Q-body was 546 nm, and the maximum fluorescence peak of the emission wavelength appeared at 584 nm, corresponding to the TAMRA fluorescent dye (Figure 3H). The increase in fluorescence intensity of ticagrelor detected by 2452A and 152T Q-body was 3.50-fold and 3.40-fold, respectively (Figures 3G,I). The LOD of assays with the 2452A and 152T Q-body was 1.95 and 2.15 pg/mL, and the EC_50_ was 36.75 and 81.8 pg/mL, respectively (Table 1).

**TABLE 1:** Increase in fluorescence intensity, LOD, and EC_50_ of 2452A and 152T Q-body detected in PBS or spiked serum.

Background solution	In PBS						In spiked serum					
Q-body	2452A Q-body			152T Q-body			2452A Q-body			152T Q-body		
Parameters	F.I.^a^ increase (fold)	LOD (pg/mL)	EC_50_ (pg/mL)	F.I.^a^ increase (fold)	LOD (pg/mL)	EC_50_ (pg/mL)	F.I.^a^ increase (fold)	LOD (pg/mL)	EC_50_ (ng/mL)	F.I.^a^ increase (fold)	LOD (pg/mL)	EC_50_ (ng/mL)
Ticagrelor	3.5	1.95	37.75	3.4	2.15	81.75	8.7	5.45	5.2	4.2	2.65	2.1
TAM	5.3	3.55	550	3.3	1.95	218.15	5.3	3.4	7.1	4.2	2.45	9.65
TIM	1.5	-	-	1.1	-	-	1.9	-	-	1.1	-	-
Tic:TAM (3:1)	-	-	-	-	-	-	7.6	4.65	8.15	4.2	2.75	3.0

^a^ F.I.: fluorescence increase.

**FIGURE 4:**
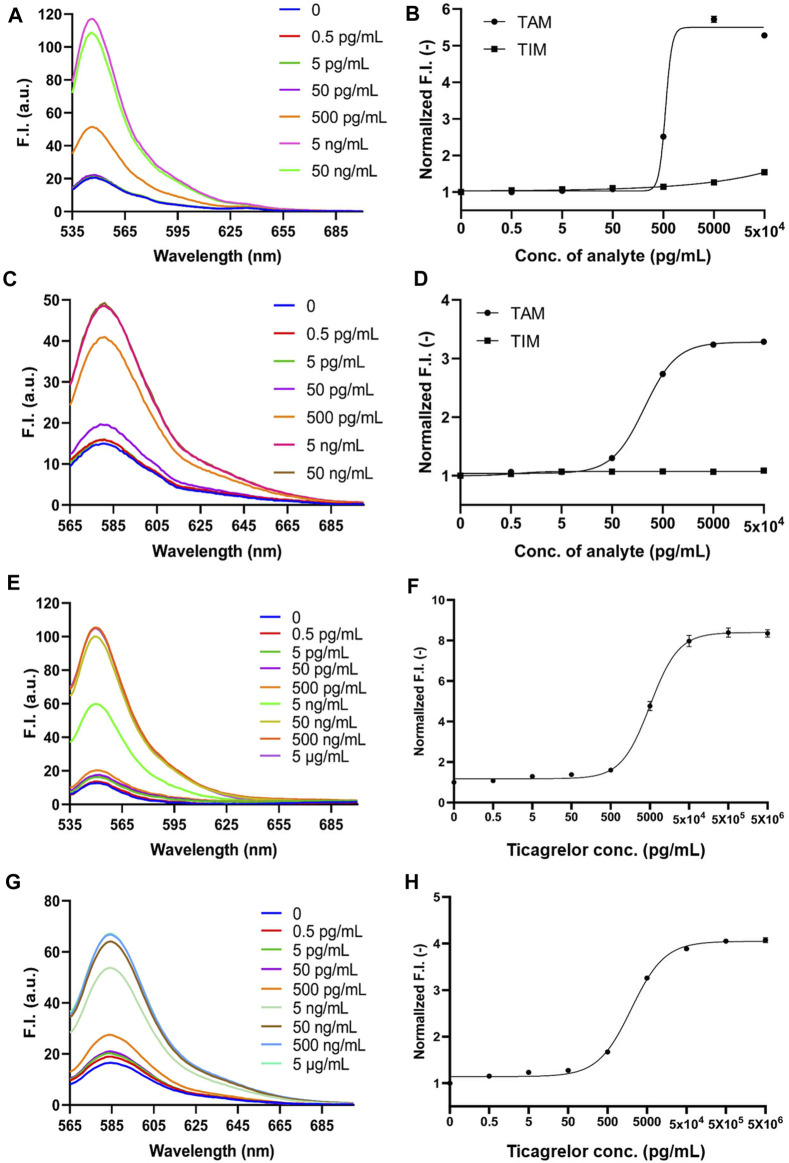
2452A and 152T Q-body detection of TAM and TIM in PBS and ticagrelor in spiked serum. **(A)** Spectra of 2452A Q-body after addition of gradient-diluted TAM with PBS as background; **(B)** Stoichiometric-response curves for detection of TAM and TIM in PBS by 2452A Q-body; **(C)** Spectra of 152T Q-body after addition of gradient-diluted TAM with PBS as background; **(D)** Stoichiometric-response curves for detection of TAM and TIM in PBS by 152T Q-body; **(E)** Spectra of 2452A Q-body after addition of a gradient dilution of ticagrelor with spiked serum as background; **(F)** 2452A Q-body assay stoichiometry-response curve for ticagrelor in spiked serum; **(G)** Spectra of 152T Q-body after addition of a gradient dilution of ticagrelor with spiked serum as background; **(H)** 152T Q-body assay stoichiometric response curve for ticagrelor in spiked serum. F.I: fluorescence intensity; Error bars, ±1 S.D (*n* = 3).

The fluorescent spectra of the 2452A and 152T for the detection of TAM are shown in Figures 4A, C. The fluorescence intensity increases of 2452A and 152T Q-body were 5.3-fold and 3.3-fold for the detection of TAM, with LODs of 3.55 and 1.95 pg/mL, and EC_50_ of 550 and 218.15 pg/mL for TAM, respectively (Figures 4B, D; Table 1). We also tested the detection effect of Q-bodies on the inactive metabolite TIM, and the results showed that TIM was basically unable to enhance the fluorescent intensity of 2452A and 152T Q-bodies with 1.1 to 1.5-fold. Besides TIM, an ATPase activity inhibitor digoxin that has a similar molecular weight size compared to ticagrelor was used as a control, and the results showed that digoxin also failed to increase the fluorescence intensity of 2452A and 152T Q-bodies (Figures 3G, I). These results indicate that the 2452A and 152T Q-bodies were highly specific for ticagrelor and TAM. The fluorescence growth intensities of 2452A and 152T Q-body in PBS were 3 to 5-fold much lower compared to the Q-body denaturation assay which may be due to the solubility of ticagrelor and TAM in PBS which did not reach the upper limit of detection of Q-body.

### 3.7 Detection of ticagrelor and TAM in serum

**FIGURE 5:**
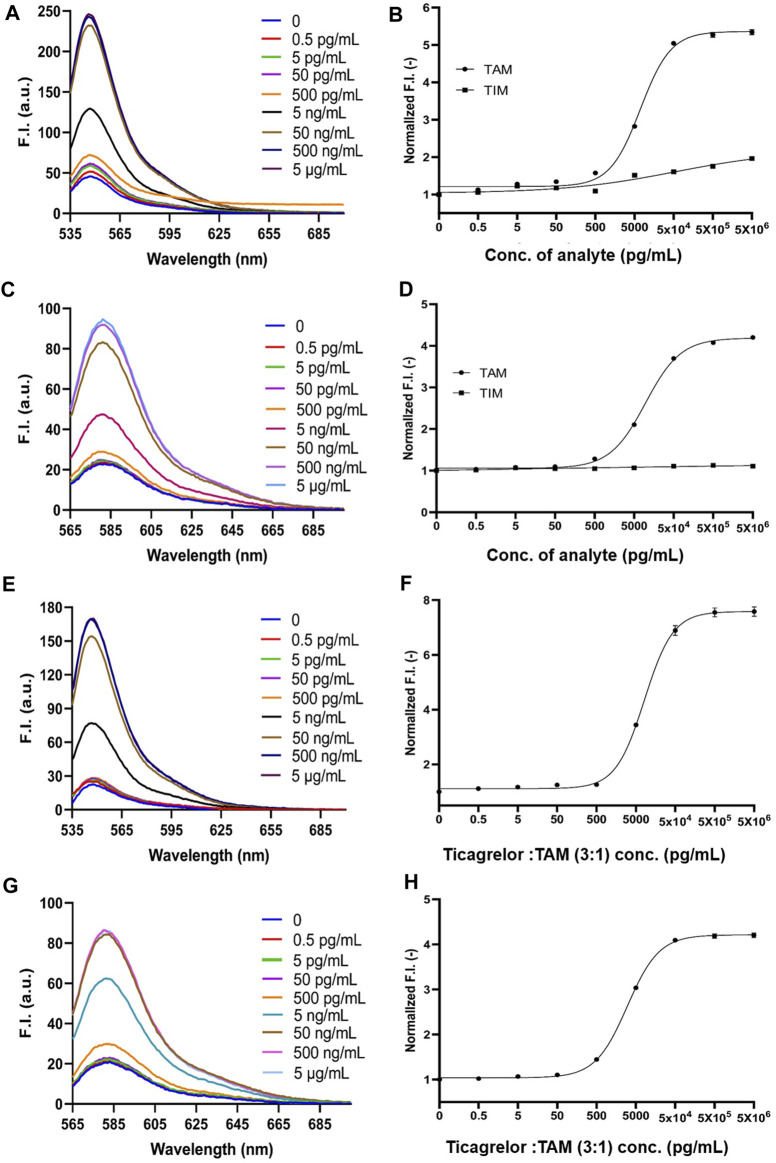
2452A and 152T Q-body detection of TAM, TIM, and the mixture of ticagrelor and TAM (3:1) in spiked serum. **(A)** Spectra of 2452A Q-body after addition of gradient-diluted TAM with spiked serum as background; **(B)** 2452A Q-body assay stoichiometry-response curve for TAM and TIM in spiked serum; **(C)** Spectra of 152T Q-body after addition of gradient-diluted TAM with spiked serum as background; **(D)** 152T Q-body assay stoichiometry-response curve for TAM and TIM in spiked serum; **(E)** Spectra of 2452A Q-body after addition of gradient diluted ticagrelor: TAM (3:1) with spiked serum as background; **(F)** Stoichiometric response curve of 2452A Q-body for detection of ticagrelor: TAM (3:1) in spiked serum; **(G)** Spectra of 152T Q-body after addition of a gradient dilution of ticagrelor: TAM (3:1) with spiked serum as background; **(H)** Stoichiometric response curve of 152T Q-body for detection of ticagrelor: TAM (3:1) in spiked serum. F.I: Fluorescence intensity; Error bars, ±1 S.D (n = 3).

Since ticagrelor and TAM are more soluble in serum than in PBS, we increased the maximum concentration to 5 μg/mL (Yang et al., 2022). The detection started as low as 0.5 pg/mL and increased in a 10-fold concentration gradient; the fluorescence intensity no longer increased when ticagrelor was added to 5 μg/mL. The fluorescent spectra of 2452A and 152T for the detection of ticagrelor in serum are shown in Figures 4E, G. The fold increase in fluorescence intensity of ticagrelor detected by 2452A and 152T Q-body was 8.7-fold and 4.2-fold (Figures 4F, H), with LOD of 5.45 and 2.65 pg/mL, and EC_50_ of 5.2 and 2.1 ng/mL, respectively (Table 1). The fluorescent spectra of 2452A and 152T for the detection of TAM in serum were also detected and shown in Figures 5A, C. The increase in fluorescence intensity of TAM detected by 2452A and 152T Q-body was 5.3-fold and 4.2-fold (Figures 5B, D), the LOD was 3.4 and 2.45 pg/mL, and the EC_50_ was 7.1 and 9.65 ng/mL, respectively (Table 1). Compared to the detection in PBS, the fluorescence intensity increase of these two Q-bodies was larger, indicating an increase in the detection range of ticagrelor and TAM, but their LOD and EC_50_ were relatively increased, suggesting a decrease in the sensitivity of the assay. On one hand, serum improves the solubility of ticagrelor and TAM, but on the other hand, serum contains substances such as hemoglobin, whose own color affects the detection, thus decreasing the sensitivity of Q-body detection (Supplementary Figure S1). The fluorescence intensity of 2452A and 152T Q-body reached 8.69 and 4.23-fold of the initial fluorescence intensity when 5 μg/mL of ticagrelor was added to the spiked serum, which was approaching the fluorescence intensity increase in GdnHCl/DTT denaturant (11.32-fold and 5.06-fold). Therefore, the 5 μg/mL almost reached the upper limit of detection.

### 3.8 Anti-ticagrelor Q-body for the detection of the mixture of ticagrelor and TAM in serum

Ticagrelor will undergo metabolism in a patient’s body and produce TAM, with a ratio of 3:1 between ticagrelor and TAM (Teng et al., 2010). Therefore, we used the mixture of ticagrelor:TAM (3:1) as a detection target in spiked serum. The fluorescent spectra of 2452A and 152T for the detection of ticagrelor in serum are shown in (Figures 5E,G). The maximum fluorescence intensity increase of 2452A and 152T Q-body was 7.6 and 4.2-fold (Figures 5F,H), with a LOD of 4.65 and 2.75 pg/mL, and EC_50_ of 8.15 and 3.0 ng/mL, respectively (Table 1). The increase in fluorescence intensity of 2452A Q-body for ticagrelor was higher than that of 152T Q-body, indicating that 2452A Q-body was more accurate in detecting ticagrelor in high concentration, while the lower LOD of 152T may indicate higher sensitivity of 152T. In addition, we performed recovery experiments using human serum samples spiked with ticagrelor:TAM (3:1) at concentrations of 0.3 ng/mL, 3 ng/mL, and 30 ng/mL. The recoveries for 2452A Q-body were 94.0%, 96.32%, and 94.67%, respectively, while the recoveries for the 152T Q-body were 90.73%, 87.1%, and 88.71%, respectively (Table 2). The recovery rate of 2452A Q-body was slightly higher than 152T Q-body, indicating that 2452A Q-body was more accurate compared to 152T Q-body in the detection serum sample.

**TABLE 2:** The recovery rate of 2452A and 152T Q-bodies in ticagrelor:TAM (3:1) spiked human serum samples.

2452A Q-body			152T Q-body		
Spiked conc. (ng/mL)	Measured concentration (ng/mL)	Recovery rate (%)	Spiked conc. (ng/mL)	Measured concentration (ng/mL)	Recovery rate (%)
0.3	0.28	94.0	0.3	27.22	90.73
3	2.89	96.32	3	2.61	87.1
30	28.4	94.67	30	26.62	88.74

Recovery rate: (measured concentration/spiked conc.) × 100%.

In conclusion, both the 2452A and 152T Q-bodies effectively detected ticagrelor and TAM in both PBS and serum, with the 2452A Q-body demonstrating slight advantages, particularly in serum detection. However, the 152T Q-body has higher excitation and emission wavelengths, which may provide advantages in detecting samples with stronger background fluorescence intensities (Supplementary Figure S1).

## 4 Discussion

Ticagrelor, a platelet agglutination inhibitor, poses a risk of tissue hemorrhage when taken, especially for patients who have ticagrelor in their system and undergo surgery, which can lead to intraoperative and postoperative hemorrhage, threatening the patients’ lives (Storey et al., 2010). Clinical assays showed that after patients received 90 mg of ticagrelor twice daily each time for 4 weeks, the lowest to highest plasma concentrations of ticagrelor and TAM were between 200–750 ng/mL and 100–250 ng/mL, respectively (Angheloiu et al., 2017). Both ticagrelor and TAM concentrations are within the detection range of the 2452A and 152T Q-bodies that were prepared in this study. In several studies, plasma concentrations of ticagrelor and TAM were analyzed using LC-MS and resulted in the LOD of approximately 1.25 ng/mL, respectively (Teng et al., 2010; Chae et al., 2019; Danielak et al., 2019; Xue et al., 2021). The LOD of 2452A and 152T Q-body for ticagrelor in 10% serum was 5.45 and 2.65 pg/mL, and the LOD for TAM was 3.40 and 2.45 pg/mL, which indicates the LODs of 54.5, 26.5, 34.0, and 24.5 pg/mL in patient serum, respectively. The LOD of 2452A and 152T Q-body for ticagrelor and TAM was significantly lower compared with that of LC-MS, which rapidly and sensitively detected lower concentrations of ticagrelor in the blood.

Both 2452A and 152T Q-body can detect ticagrelor and TAM, but the 2452A Q-body shows a higher multiplicative increase in fluorescence intensity after denaturation and addition of ticagrelor or TAM, which may be attributed to the fact that ATTO is more readily quenched by amino acids of the antibody than TAMRA. The 152T Q-body exhibited a lower LOD in serum than the 2452A Q-body, potentially due to the fact that the background fluorescence of the serum is mainly concentrated in the ATTO peak band (Supplementary Figure S1A), with less impact on the TAMRA peak band (Supplementary Figure S1B). Despite the relatively high LOD of the 2452A Q-body, it demonstrated a wide detection range. Consequently, these two Q-bodies can complement each other and play a crucial role in ticagrelor detection. The affinity of the antibody is a key factor affecting the sensitivity of the immunoassay. Therefore, we replaced the prokaryotic-expressed Fab or scFv with eukaryotic-expressed full-length antibody IgG, which has a more stable structure and activity than Fab or scFv. Moving forward, more sensitive Q-bodies can be developed by artificially evolving antibodies through OS selection technology or phage display technology (Iwai et al., 2010).

We also prepared BSA-Tic for ELISA to detect the antigen-binding activity of full-length antibodies. However, BSA-Tic is not a potential platelet aggregation inhibitor. It may be due to the spatial hindrance caused by structural changes after ticagrelor was bound to BSA and prevented it from binding properly to the ADP-binding site of P2Y_12_. This suggests that the conjugation of proteins may inhibit the activity of small molecules. In fact, many antibody-conjugated drugs often necessitate the liberation of small molecules from antibodies in order to exert their molecular functions (Tsuchikama and An, 2018). Additionally, the BSA-Tic can be used as a good competitive substrate in the competitive ELISA detection of ticagrelor and also in various biochemical experiments.

In this study, spiked serums were used to simulate the clinical detection of ticagrelor in practice. In future studies, the direct use of clinical samples will enhance the applicability of ticagrelor Q-bodies. Moreover, the emission wavelengths of ATTO and TAMRA are below 600 nm, which may be affected by background fluorescence in serum and partly impact the sensitivity of Q-bodies (Supplementary Figure S1). Therefore, it is necessary to develop infrared fluorescent molecules that can be applied to prepare Q-bodies.

In summary, we have successfully developed immunofluorescent sensors for rapid detection of ticagrelor and its active metabolite, TAM. These sensors demonstrate excellent stability, reproducibility, and rapid detection speed. They can provide timely data as a fundamental tool for monitoring ticagrelor and TAM levels in patients’ blood, assisting doctors in determining further treatment plans. We believe that this detection technique holds great promise for clinical diagnosis, offering valuable preoperative guidance for patients with ACS receiving ticagrelor. Furthermore, the design, synthesis, and application of ticagrelor-NHS ester lay the foundation for its broader application in basic medicine and biochemical research.

## Data Availability

The original contributions presented in the study are included in the article/Supplementary Material, further inquiries can be directed to the corresponding authors.
