# Supplementary material for: Fluorescence quenching-based immunological probe for ticagrelor monitoring
Source: Front Bioeng Biotechnol. 2023 Nov 28;11:1295406. doi: 10.3389/fbioe.2023.1295406 (PMC10715258; doi:10.3389/fbioe.2023.1295406)
Supplement: Supplementary file 1 [file DataSheet1.docx]

**Fluorescence quenching based immunological probe for ticagrelor monitoring**

**Supplemental Data**

Shengshuo Zhang^1,2^, Yueqing Cheng^1^, Yujie Gao^1^, Yujie Zou^1^, Weiling Xiao^3^, Tianyi Li^4^, Mei Li^1^, Bowen Yu^3*^, Jinhua Dong^2,5*^

^1^*School of Life Science and Technology,* *Weifang Medical University, Weifang, China*

^2^*School of Rehabilitation Sciences and Engineering, University of Health and Rehabilitation Sciences, Qingdao, China*

^3^*School of Basic Medical Sciences, Weifang Medical University, Weifang, China*

^4^*School of Stomatology, Weifang Medical University, Weifang, China*

^5^*International Research Frontiers Initiative, Tokyo Institute of Technology, Yokohama, Japan*

^*^Corresponding authors

Jinhua Dong, [jhdong@uor.edu.cn](mailto:jhdong@uor.edu.cn)

Bowen Yu, yubowen@wfmc.edu.cn

**
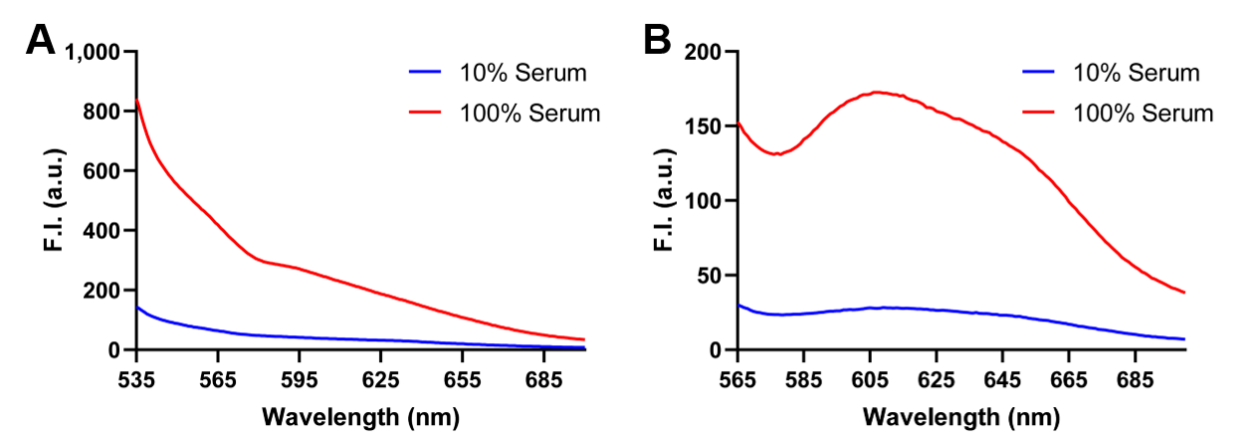
**

**Fig. S1. Serum background testing.** (A) PBS containing 10% serum and 100% serum were detected at 520 nm as excitation wavelength and 535 nm as emission wavelength; (B) PBS containing 10% serum and 100% serum were detected at 546 nm as excitation wavelength and 565 nm as emission wavelength; Mean (n = 3), ±1 S.D.

.

**Table S1** Primers used in this study.

| **Primer name** | **Sequence (5′ -3′)** | **Length (bp)** |
| --- | --- | --- |
| 5ALBLfor | GGGTGAGGGCCGGTACCAGTAAACAGTGCTCTAACGAGACAAGC | 44 |
| 5ALBLback | TGCTAGCAAGCTTCTCGAGTTAGTGATGGTGATGGTGGTGCTCAGTGGGGGCGACTG | 57 |
| 5ALBHfor | GGGTGAGGGCCGGTACCGCTCAGATTGAGGTGAATTGCTCC | 41 |
| 5ALBHback | CCCAGGAGTTCAGGTGCGCTCTTGGGCTCCACCC | 34 |
| 5ALBFcfor | GCACCTGAACTCCTGGGG | 18 |
| 5ALBFcback | CCTCTCCCTGTCTCCGGGTAAATAACTCGAGAAGCTTGCTAGCA | 44 |
| 5ALBMutantLfor | GATATCTCTTTGTCCGCGGGGCTGTTCGGCGG | 32 |
| 5ALBMutantLback | CCCCGCGGACAAAGAGATATCCCAGGTCCCGCAGTAGT | 38 |
| 5ALBMutantHfor | GGGGAGCCACCTCTACGACTTCTGGAGCGCCAGC | 34 |
| 5ALBMutantHback | CTCCAGAAGTCGTAGAGGTGGCTCCCCCTGGCGCA | 35 |

**Table S2** Ratio of fluorescence intensity of 8 Q-bodies in GdnHCl/DTT to initial fluorescence intensity in PBS.

| **Samples**  **Dye** | **MEDI2452** | **IgG 72** | **IgG 152** | **IgG 162** |
| --- | --- | --- | --- | --- |
| ATTO | 11.32 | 2.99 | 7.32 | 2.56 |
| TAMRA | 2.61 | 1.40 | 5.06 | 2.81 |

**Table S3** CDR3 sequences of the four antibodies.

| **Antibody** | **VH CDR3** | **VL CDR3** | **Vectors** |
| --- | --- | --- | --- |
| MEDI2452 | GSFDYYFWSASHPPNDALAI | GTWLYDRAVGL | pMlink-5ALB-H and pMlink-5ALB-L |
| IgG 152 | GSHLYDFWSASHPPNDALAI | GTWLYDRAVGL | pMlink-5ALB-HM and pMlink-5ALB-L |
| IgG 72 | GSHLYDFWSASHPPNDALAI | GTWDISLSAGL | pMlink-5ALB-HM and pMlink-5ALB-LM |
| IgG 162 | GSFDYYFWSASHPPNDALAI | GTWDISLSAGL | pMlink-5ALB-H and pMlink-5ALB-LM |

Difference in tyrosine between the heavy chain CDR3 regions and the light chain CDR3 regions of the four antibody fragments was underlined.
